# Risk burdens of modifiable risk factors incorporating lipoprotein (a) and low serum albumin concentrations for first incident acute myocardial infarction

**DOI:** 10.1038/srep35463

**Published:** 2016-10-17

**Authors:** Qin Yang, Yong-Ming He, Dong-Ping Cai, Xiang-Jun Yang, Hai-Feng Xu

**Affiliations:** 1 Division of Cardiology, Department of Medicine & Therapeutics, The Chinese University of Hong Kong, 30 Ngan Shing St, Shatin, New Territories, Hong Kong; 2 Division of Cardiology, the First Affiliated Hospital of Soochow University, 188 Shizi Ave., Gusu District, Suzhou City, Jiangsu Province, 215006, P. R. China; 3 Healthcare Center for Shishan Street Community, Suzhou, 211 Yushan Rd., Huqiu District, Suzhou City, Jiangsu Province,215011, P. R. China.

## Abstract

Risk burdens of modifiable risk factors incorporating lipoprotein (a) (Lp(a)) and low serum albumin (LSA) concentrations for first incident acute myocardial infarction (AMI) haven’t been studied previously. Cross-sectional study of 1552 cases and 6125 controls was performed for identifying the association of risk factors with first incident AMI and their corresponding population attributable risks (PARs). Modifiable risk factors incorporating LSA and Lp(a) accounted for up to 92% of PAR for first incident AMI. Effects of these risk factors were different in different sexes across different age categories. Overall, smoking and LSA were the 2 strongest risk factors, together accounting for 64% of PAR for first incident AMI. After multivariable adjustment, Lp(a) and LSA accounted for 19% and 41%, respectively, and together for more than a half (54%) of PAR for first incident AMI. Modifiable risk factors incorporating LSA and Lp(a) have accounted for an overwhelmingly large proportion of the risk of first incident AMI, indicating most first incident AMI is preventable. The knowledge of risk burdens for first incident AMI incorporating Lp (a) and LSA may be beneficial for further reducing first incident AMI from a new angle.

Mortality from coronary artery disease (CAD) has declined steadily in industrialized countries in past two to three decades, undoubtedly owing to vigorous risk factor intervention and to improved treatment^1^,^2^,^3^,^4^. Unfortunately, the mortality from cardiovascular diseases (CVDs), as the leading cause of death, has increased significantly in past two decades in China, almost being twice that of malignancies in 2013^5^. In China, the mortality from CAD increased from 55.7/100,000 in 1990 to 70.1/100,000 in 2010, with a 31.6% increase^6^. Risk factor modification has been the cornerstone for prevention and therapy of CVDs. Current knowledge of prevention and treatment of CVDs is mainly derived from Caucasian populations. The association magnitude and prevalence of a risk factor vary in different regions in different ethnics: e.g. blood pressure elevation triples the risk of CVDs in Chinese, equivalent to synergistic effects of any three other risk factors, which is different from reported data from Caucasians^7^. The INTERHEART study also admitted approach to prevention of CAD varied in different subgroups based on the prevalence of individual risk factors^8^. The INTERHEART China study unfortunately failed to present risk burdens of individual risk factors in different sexes in age categories^9^. Lp(a), a recently identified risk factor for CAD, is on average far lower in serum concentrations in Chinese than in Caucasians^10^,^11^. Therefore, cardiologists are unsure to what extent these findings apply in different sexes in varying age categories in Chinese Han ethnic population. LSA, an important risk factor for CVDs both in Caucasians^12^,^13^ and in Chinese^14^, hasn’t been studied to know about its importance in CAD. Additionally, higher Lp(a) has been proposed as a residual risk for cardiovascular diseases^15^. Therefore, the knowledge of risk burdens for CAD in terms of modifiable risk factors incorporating Lp (a) and LSA concentrations may be important for elimination of residual cardiovascular risks. The current study will calculate the population attributable risks (PARs) of modifiable risk factors incorporating LSA and Lp(a) levels, which will conduce to policymakers’ knowledge of varying risk burdens and to further reduction of first incident AMI from a new angle.

## Methods

### The database

The database of CCSSSCC has been described elsewhere^10^. Briefly, this desk top database file system includes around 35,000 consecutive patients admitted into this Division of Cardiology ever since Jan. 1, 2002. All patient records were anonymized and de-identified, and the informed consents were waived by Institute Review Boards (IRB) before analysis due to the retrospective nature of the study. The study protocol was approved by the IRB of Soochow University. The current study is in line with the principles outlined in the Declaration of Helsinki.

### Patients

Patient selection has been described in details elsewhere^10^. Briefly, all patients admitted between Jan. 1, 2010 and Dec. 31, 2013, were included for potential analysis. The former 5 exclusion criteria were the same as those listed in the literature by Cai *et al*.^10^. In the current study, patients with initial ischemic heart disease or prior CAD defined in the literature by Cai *et al*.^10^ were also excluded. For a patient with multiple hospitalizations, we only collected the first admission data. For a patient with multiple lab exams during hospital stay, we only collected the first-measured lab results.

Information on demographic factors, lifestyles, vital signs, comorbidities, lab exams, medications at discharge, lipid profiles, and coronary angiography was collected. The data on height were missing in 5.85% of enrollee; body weight, in 10.62%; marriage status, in 0.77%; systolic blood pressure, in 0.55%; diastolic blood pressure, in 0.61%; heart rate, in 0.71%; smoking status, 3.61%; drinking status, in 3.64%; and hemoglobin, in 4.46%. Total protein, albumin, creatinin, ALT, and AST were missing around in 0.64% of participants.

### Definitions, diagnoses and grouping

Smoking status, drinking status, body mass index, CAD, hypertension, thyroid dysfunction, and kidney dysfunction have been defined in details elsewhere^10^. Chief complaints, cardiac biomarker exams, coronary angiography, echocardiography, treadmill excise test, Holter monitoring, and electrocardiography were used for diagnosing CAD. The CAD diagnosis included 3 categories of ischemic heart disease, prior CAD and first incident AMI, which were defined in the literature by Cai *et al*.^10^.

The first incident AMI was defined as first ever onset acute myocardial infarction diagnosed in this division in line with the universal myocardial infarction definition^16^, without a past history of myocardial infarction. Therefore, a total of 7647 patients met the inclusion criteria, amongst whom, 1522 first incident AMI patients were used as cases, and 6125 non-CAD patients admitted during the same period served as non-CAD controls. The diagnoses in the non-CAD controls at discharge were 55% with hypertension, 12% with diabetes mellitus, 18% with paroxysmal supraventricular tachycardia, 13% with atrial fibrillation/atrial flutter, and 29% with the miscellaneous.

### Lab measurements

The blood samples were drawn after 8 h fasting on the 2^nd^ day morning after admission. The diagnostic reagent kits were purchased from Sekisui Co. Ltd., to quantify total protein, albumin, creatinine, ALT, AST, hemoglobin, TC, TG, Lp (a), apo A, apo B, LDL-C, and HDL-C according to the manufacturer’s specifications. Olympus AU5400 analyzer was employed for the analysis. Calibrators, in compliance with the IFCC PRM-2, supplied by Sekisui Diagnostic Ltd., were used for calibrating the examination results. The intra-assay and inter-assay CVs for biochemical markers were within acceptable ranges pre-specified by the manufacturer. Additionally, 4-time-one-year externally quality assessment (EQA), presided by the Clinical Lab Examination Center of Health Ministry of PR China, has been done to ensure the reliability of the lab examinations. A blood sample with any measurements beyond the assay ranges would be routinely diluted 1:10. Thus, we did our best to ensure that all biomarkers were kept in the security range of the assay.

The Lp(a) measurement method has been described in details elsewhere^10^. Briefly, the Lp (a) concentrations were determined using the latex-enhanced immunoturbidimetric diagnostic reagent kits. The assay range is 10–1000 mg/L. The blood samples with the Lp (a) >1000 mg/L were routinely diluted 1:10. Thus, the Lp (a) concentrations up to 10,000 mg/L were within the security range of the assay and wouldn’t mistakenly be considered as a low level due to antigen excess. Lp (a) protein calibrator, in line with the IFCC PRM-2, provided by Sekisui Co. Ltd., has been used to calibrate the Lp (a) examination results. The intra-assay and inter-assay coefficients of variation for Lp (a) were 2.5% and 3.11%, respectively.

### Statistical analysis

Shapiro-Wilk test was hired for testing normality for continuous variables. As all the continuous variables in the current study failed to obey the distribution of normality, we thus expressed them as median (inter quartile range, IQR) and compared using Kruskal-Wallis rank test. Categorical variables were expressed as frequencies and percentages, and compared using Likelihood-ratio *Chi* squared test. Missing values for continuous variables were filled in with the corresponding medians, and for categorical ones, randomly filled in according to the corresponding proportion of the distribution of that variable.

The quantiles of all continuous variable were calculated on the basis of the non-CAD controls. The cutoffs used in both sexes divided all participants into thirds. Tirtiles of continuous variables were incorporated into regression models as design variable.

Unconditional logistic regression was performed for model fitting for odds ratios of risk factors for first incident AMI. Model 1: crude OR, with no adjustment for any risk factors; model 2: partially adjusted OR, with adjustment for unmodifiable age and sex; model 3: fully adjusted OR, with adjustment for Lp(a), drinking, smoking, hypertension, diabetes mellitus, BMI, LDL-C, HDL-C, TG, ALB, and ischemic stroke, exam year, Cr and hemorrhagic stroke plus age and sex. Variance inflation factor (VIF) was used for quantifying the potential presence of multicollinearity among covariates in the fully adjusted model. A VIF > 5 was considered for the presence of collinearity according to a common rule of thumb. Thus, the ratio of apo A to apo B, apo B, apo A, and TC were removed from the model fitting. Estimates of ORs and 95% CIs were reported for risk factors. Statistical analysis and graphics were completed with STATA 13.0. 2-tailed P < 0.05 was statistically significant. The given PARs and 95% CIs were calculated for every risk factor and every combination of risk factors based on the same unconditional logistic regression model.

For a simple exposure and disease, with no adjustment for confounding, the PAR calculation was performed with the formula (1) below^17^. Combined PAR calculation adjusted for confounding was performed with the formula (2) based on the multivariate adjusted logistic regression model^18^. The interactive risk attributable program software (IRAP 2.0, US National Cancer Institute, 2002) was used to do all the PARs calculations^19^.


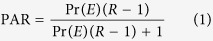


where Pr(*E*) is probability of exposure to the risk factor and *R* is the relative risk of the disease in exposed versus unexposed individuals.

A PAR adjusted for confounding is given by:


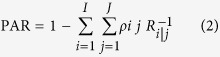



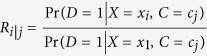



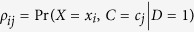


X is the exposure level, C is the confounder levels, and D is disease status (D = 0, disease is absent; D = 1, disease is present). For variance estimates, the reader refers to Benichou and Gail as the derivations and formulae are complex^18^.

## Results

**Figure 1:**
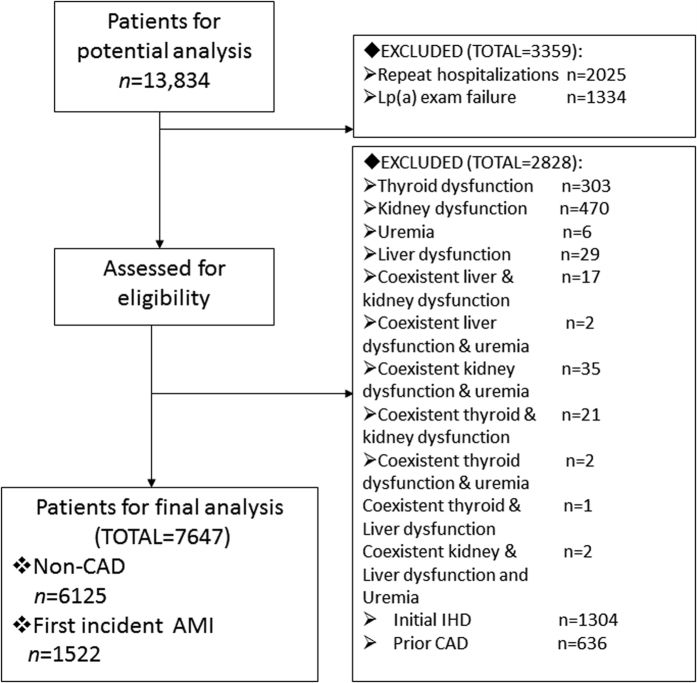
The flow chart of patient selection.

Patient selection has been described in details elsewhere. Briefly, a total of 13,834 consecutive patients were included for potential analysis, amongst whom, 888 were excluded because of thyroid, kidney, liver dysfunction or uremia or coexistent entities mentioned above; 1334, because of failure to examine Lp (a); and 2025, because of repeat hospitalizations. In addition, 636 patients with prior CAD and 1340 with ischemic heart disease were also excluded in the current study. Details were seen in flow chart in Fig. 1.

### Demographic and baseline data of study subjects

As expected, risk factors, such as male sex, diabetes, hypertension, aging, higher BMI, smoking, dyslipidemia, etc. associated with CVDs were more likely to be with first incident AMI. Systolic and diastolic blood pressures were overall lower in AMI patients than in controls, indicating systematic affection by CAD. Cardiac biomarkers, such as ALT and AST, were increased while total protein and albumin levels were decreased in AMI patients. Up to ~91% of first incident AMI patients and ~28% of non-CAD controls received the CAG examination. See Table 1.

### Overall effect of risk factors

Table 2 provides overall odds ratios for individual risk factors examined in the current study, without adjustments (crude model), adjusting only for age and sex (partial model), and by multivariate adjustment for all risk factors (full model). Age and male sex are 2 unmodifiable risk factors, accounting for 54.75% and 22.22%, respectively, of PAR for first incident AMI. After multivariate analysis, smoking and LSA concentrations were the 2 strongest risk factors, followed by body mass index, LDL-C, diabetes mellitus, Lp(a), and HDL-C. Interestingly and also unexpectedly, both TG and hypertension were overall not significantly associated with first incident AMI after progressive multivariate adjustments. Alcohol abstinence and ischemic stroke seem to be protective risk factors for first incident AMI, with the odds ratio of 0.64 (95% CI, 0.41–0.99) and 0.56 (95% CI, 0.43–0.75), respectively, accounting for a very small proportion of PAR for first incident AMI. See details in Table 2.

**Figure 2:**
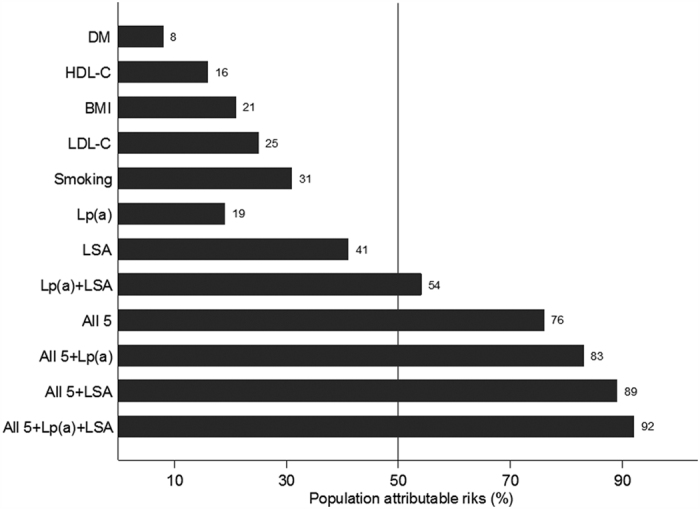
PARs of risk factors associated significantly with first incident AMI. All 5 indicated 5 conventional risk factors mentioned in this figure except Lp(a) and LSA. PAR calculations in combination or separately were all based on fully adjusted logistic regression model. Lp(a) accounted for 19% of PAR for first incident AMI; LSA, for 41%, and Lp(a) and LSA together, for 54%. The sum of separate Lp(a) and LSA PARs didn’t equal the combined PAR calculation for Lp(a) and LSA together, indicating the presence of overlap in contribution of Lp(a) and LSA. LSA and smoking were the 2 most important risk factors for AMI. The abbreviations as in Table 1.

Together, smoking, LDL-C, BMI, HDL-C, and diabetes mellitus accounted for 76.01% of PAR for first incident AMI, increasing substantially with addition of albumin, Lp (a), or both. Strikingly, LSA and Lp (a) in combination accounted for more than a half (54.47%) of PAR for first incident AMI. See details in Table 3 and in Fig. 2.

### Risk by sex

In women, LSA and LDL-C were the 2 strongest risk factors, followed by diabetes mellitus, body mass index, Lp (a), triglycerides, and hypertension. In contrast, smoking and LSA were the 2 strongest risk factors, followed by BMI, LDL-C, DM, Lp (a), and HDL-C in men. Drinking abstinence or ischemic stroke seemed to be two protective factors for first incident AMI in men, but accounting for a very small proportion (1%) of PAR. Smoking increased the risk of first incident AMI in men, but not in women while hypertension increased the risk of first incident AMI in women, but not in men. Greater risk burdens were with hypertension, diabetes, LSA, Lp (a), LDL-C and triglycerides in women than in men. By contrast, smoking and HDL-C increased more risk of first incident AMI in men than in women. Similar risk burdens were recorded with body mass index in both sexes. See details in Table 4.

**Figure 3:**
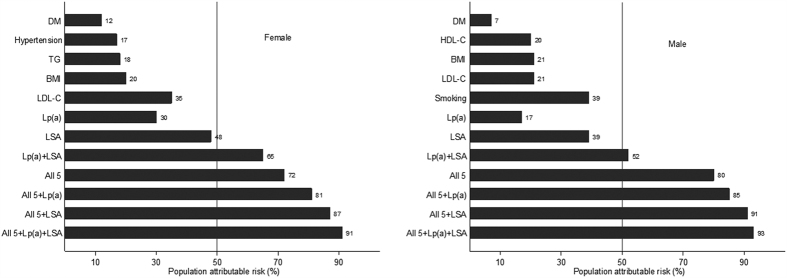
PARs of risk factors associated significantly with first incident AMI by sex. LDL-C and LSA were the 2 most important risk factors for AMI in females. Significant association of hypertension and TG with AMI were exclusively seen in females. In contrast, smoking and LSA were the 2 most important risk factors for AMI in males. Significant association of HDL-C and smoking with AMI were exclusively seen in males. The abbreviations as in Table 1.

In women, LDL-C, DM, BMI, TG, and hypertension together accounted for 71.9% of PAR for first incident AMI, increasing substantially with addition of LSA, Lp (a) or both. The PAR for LSA and Lp (a) accounted for almost two-thirds of PAR for first incident AMI. In men, smoking, BMI, LDL-C, DM, and HDL-C accounted for 80.22% of PAR for first incident AMI, increasing substantially with addition of LSA, Lp (a), or both. LSA and Lp (a) together accounted for about a half of PAR for first incident AMI. See details in Table 3 and in Fig. 3.

### Risk by sex and age

In age category ≤55 y, triglycerides was surprisingly the strongest risk factor, followed by LSA and BMI in women while LSA and smoking were the 2 strongest risk factors, followed by BMI, diabetes mellitus, LDL-C, Lp(a), and HDL-C in men. Triglycerides and BMI together accounted for 84.76% of PAR for first incident AMI, increasing to 89.58% with addition of LSA in women. By contrast, smoking, BMI, LDL-C, DM, and HDL-C together accounted for 82.68% of PAR for first incident AMI, increasing substantially with addition of LSA, Lp (a), or both in men. LSA and Lp (a) together accounted for 47.77% of PAR for first incident AMI in men.

In age category 55~67 y, LSA and Lp (a) were two strongest risk factors, followed by DM, TG, and LDL-C in women while smoking and LSA were the 2 strongest risk factors, followed by DM, BMI, LDL-C, HDL-C, and Lp (a) in men. DM, TG, and LDL-C together accounted for 70.09% of PAR for first incident AMI, increasing substantially with addition of LSA, Lp (a), or both in women. LSA and Lp (a) together accounted for 71.99% of PAR for first incident AMI in women. By contrast, smoking, DM, BMI, LDL-C, and HDL-C together accounted for 82.8% of PAR, increasing substantially with addition of LSA, Lp (a), or both in men. LSA and Lp (a) together accounted for 48.45% of PAR for first incident AMI in men.

In age category >67 y, LSA and BMI were the two strongest risk factors, followed by LDL-C, DM, and Lp (a) in women while smoking and BMI were the 2 strongest risk factors, followed by LDL-C, LSA, HDL-C, DM, and Lp(a) in men. BMI, LDL-C, and DM together accounted for 54.78% of PAR for first incident AMI, increasing substantially with addition of LSA, Lp (a) or both in women. LSA and Lp (a) contributed to 63.28% of PAR for first incident AMI in women. By contrast, smoking, BMI, LDL-C, DM, and HDL-C together accounted for 75.93% of PAR for first incident AMI, increasing substantially with addition of LSA, Lp (a), or both in men. LSA and Lp (a) together accounted for a half of PAR for first incident AMI in men. See details in Table 3 and Table 5.

## Discussion

To our knowledge, this is the first cross-sectional study ever conducted to explore the risk burdens incorporating LSA concentrations and Lp (a) for first incident AMI. The current study has demonstrated that modifiable risk factors incorporating LSA and Lp(a) account for an overwhelmingly large (up to 92%) proportion of the risk of first incident AMI, indicating that most first incident AMI is preventable. The effects of these risk factors are different in women and men, across different age categories, entailing tailored prevention strategies for different populations. Overall, smoking and LSA are the 2 strongest risk factors, together accounting for 64% of PAR for first incident AMI. After multivariable adjustment, Lp(a) and LSA have accounted for 19% and 41%, respectively, and together for more than a half (54%) of PAR for first incident AMI. Unexpectedly, hypertension is not significantly associated with first incident AMI. Alcohol abstinence and ischemic stroke seem to be protective risk factors for first incident AMI

Male sex and age, 2 unmodifiable risk factors, are significantly associated with first incident AMI in univariate analysis or after multivariable adjustment, and accounting for 55% and 22%, respectively, of PAR for first incident AMI (calculated on the univariate analysis). After adjustment for sex and age, potentially modifiable risk factors significantly associated with first incident AMI have accounted for more than 90% of PAR. Therefore, these two unmodifiable risk factors have a substantial overlap in contributions of other risk factors. That meant that risk burdens conferred by male sex and age increase could be offset by modification of unhealthy lifestyles, comorbidities, or abnormal biomarkers.

Current smoking is the strongest risk factor with the odds ratio of 3.57, accounting for one-third of PAR for first incident AMI after multivariable adjustment. When stratified by sex, the significantly strong association of current smoking with first incident AMI is exclusively seen in men, but not in women. The PAR has been affected by both odds ratio and prevalence. Current smoking in male cases has a prevalence of as high as 66% compared with only 3% in female cases, consistent with a previous study^20^. Therefore, the PAR attributable to current smoking is mainly derived from first incident AMI male patients. Smoking control campaigns should be targeted to Chinese male population. In INTERHEART study^8^, a similar PAR for AMI was reported (36% vs 31%) in Chinese/HK population albeit with an attenuated association of 2.30 compared with 3.57 reported in the current study. The attenuated association magnitude could be in part explained by combined exposure of current or former smoking in the INTERHEART study while odds ratios were reported separately for current and former smoking in the current study. The current study had a similar prevalence of current or former smoking (62.0% vs 62.2%) in cases, but had a far lower prevalence of smoking (28% vs 43%) in controls, which could account for the attenuated magnitude of association seen in the INTERHEART study. The controls in the current study were all hospitalized non-CAD patients, whose lifestyles may be more likely to be changed by disease state. By contrast, 36% of controls in the INTERHEART study were apparently healthy attendants or relatives of patients, whose unhealthy lifestyles tended to be unchanged. Therefore, the different control patient selections may in part explain the great difference in the prevalence of smoking in controls between the INTERHEART study and the current study. The decreased prevalence of smoking could explain the same decreasing trend for PARs estimates of smoking for first incident AMI successively across the age categories albeit a similar association magnitude (odds ratios: 3.53~3.59).

Greater magnitude of association and PAR of diabetes with first incident AMI was recorded in women than in men (2.13 vs 2.05, and 12% vs 7%, respectively), consistent with previous studies^8^,^20^. In the current study, BMI significantly increased the risk of first incident AMI with mid/bottom third, but not with top/bottom third, which could apparently be attributable to chance because of small sample sized cases in subgroup analysis. Risk burdens of BMI recorded in both sexes in Chinese were similar to previous reports^20^. The PAR of BMI ranged from 22–27% in Chinese men with a narrow fluctuation across age categories. However, the PARs of BMI were greater (39% and 32%, respectively) in youngest age category and in oldest age category than in middle age category in Chinese women, entailing differential approach to excess body weight in specific population.

Clinical Guidelines seek to lower LDL-C with statin therapy as their primary goals in CVDs treatment^21^,^22^. We thus only studied the risk of LDL-C rather than the ratio of Apo B/Apo A1 in the INTERHEART^8^ or TC in the Copenhagen City Heart Study and the Malmo Project^23^,^24^. The current study used bottom third of LDL-C as a reference (2.2 mmol/L) and reported a significant association with first incident AMI in middle or top third of LDL-C (OR, 1.47 and 2.27, respectively), accounting for 25% of PAR for first incident AMI. For patients with AMI, guidelines recommended the LDL-C be <1.8 mmol/L, lower than the cutoff employed in the current study^21^. Therefore, our study underestimated the risk burden of LDL-C in AMI patients. That also meant that AMI patients could benefit from further reduction of LDL-C levels through vigorous statin therapy in the current study. When stratified by sex, greater risk burden of LDL-C for first incident AMI was recorded in women than in men, consistent with previous studies^8^; when further stratified by age, greater risk burdens of LDL-C were recorded in youngest 2 age categories in both sexes albeit an unreliable PAR estimate of LDL-C in the youngest female age category because of small sample size in subgroup analysis. The greater risk burden of LDL-C seen in women and in youngest 2 age categories could be explained by the higher LDL-C levels in women and in youngest 2 age categories in the current study (data not shown).

In the current study, elevated triglycerides were significantly associated with first incident AMI exclusively seen in the youngest 2 age categories in women, but not in men. One study showed that triglyceride levels were not associated with cardiovascular mortality after adjusting for HDL-C and LDL-C while another study concluded that triglyceride levels were significantly associated with CVD mortality only in women, which was similar to our findings^25^,^26^. A meta-analysis demonstrated that elevated triglycerides were significantly associated with a 30% increased risk in men, and a 75% increased risk in women of CAD^27^. There remained a significant difference in terms of relative risks with elevated triglycerides between men and women even after adjustment for HDL-C and other risk factors^27^. Therefore, risk burdens truly vary with elevated triglycerides in both sexes.

Low HDL-C was associated with excess events and mortality in CVDs in both sexes^21^. In the current study, however, Low HDL-C levels predicted an increased risk for first incident AMI across age categories only in men, but not in women. The current study used the AMI while previous studies used CVD events and deaths as endpoints. Also, Chinese women had a 5 mg/dL higher levels of HDL-C than Chinese men did, far less than the 10 mg/dL difference seen in both sexes in Caucasians^28^. Finally, in the current study, men had a constant HDL-C level and women had a decreasing HDL-C level across the age quintiles (data not shown). In the Caucasians, HDL-C concentrations decreased in men while stayed constant in women with age^29^. These differences in terms of endpoints, baseline levels of HDL-C, and evolution of HDL-C levels may explain the different findings that only in Chinese men, were low HDL-C levels significantly associated with first incident AMI. The exact explanation warrants further study.

Most unexpectedly, the current study revealed that hypertension had no significant association with first incident AMI after multivariable adjustment. In univariate analysis, the hypertension was significantly associated with first incident AMI. This association was attenuated without significant differences (odds ratio, 1.1, 95% CI, 0.97–1.24) after adjusting for age and sex, and further attenuated (odds ratio, 1.05, 95% CI, 0.92–1.21) after adjusting for other risk factors plus age and sex, which indicated that this AMI-hypertension association could be largely explained by age and sex as well as other modifiable risk factors. Several prospective studies reported that hypertension was significantly associated with CAD, accounting for 14–38% of PAR in men and for 14–44% of PAR in women^20^,^23^,^24^. In the INTERHEART with a case-control design, hypertension conferred a significantly increased risk of acute myocardial infarction, accounting for 22.1% of PAR for AMI in Chinese^8^. Those prospective studies were population-based with a hypertension prevalence of 23–48% in men, and 21–40% in women; the INTERHEART study was case-control designed with a hypertension prevalence of 39% in cases, and 22% in controls. The current study was hospitalized patient-based with a hypertension prevalence of as high as 61% in cases, and 55% in controls, far higher than the above-mentioned studies, with a less prevalence difference, which will significantly attenuate the hypertension-AMI association. When stratified by sex, a significant association of hypertension with first incident AMI was revealed exclusively in women, but not in men, accounting for 17% of PAR for first incident AMI. In the current study, there had been a hypertension prevalence of up to 72% in cases, and 55% in controls in women. Therefore, the discrepancy in prevalence will heavily influence magnitude of association as well as PAR of a risk factor. When further stratified by age, odds ratios were weaker with hypertension and great variability was noted across age categories. This apparent variability could be attributable to a small sample sized cases in subgroup analysis. The overall ORs and PARs estimates with hypertension should be used in age categories in women.

Numerous clinical studies have demonstrated that stroke is significantly associated with subclinical (not symptomatically) CAD^30^,^31^,^32^. However, a few clinical studies have not demonstrated this association^33^,^34^. Most studies reporting the presence of association of stroke with CAD are actually with subclinical CAD, not with overt CAD as outcome^30^,^31^,^32^. In the current study, first incident AMI is treated as outcome completely different from previous studies. Therefore, the stroke-CAD association may have been overestimated and has remained inconclusive at least up till now^35^. Thus, it’s difficult to deny our findings that the prior stroke decreases the risk of the first incident AMI in spite of small event numbers in both control and case groups. A separate study is warranted for elucidation of the stroke-AMI association.

Solid evidence has demonstrated that alcohol, when consumed on a regular basis and at low volumes confers protection against cardiovascular disease, whereas regular amounts of more than 4–5 drinks daily and heavy episodic drinking have opposite effects^36^,^37^. Surprisingly, alcohol consumers in South Asia and the Middle East do not enjoy protection against myocardial infarction as compared with the rest of the world in the INTERHEART study^38^. It’s challenging to resolve this South Asia Paradox up till now^39^. In the current study, alcohol intake has also unexpectedly failed to protect against first incident AMI, and alcohol abstinence has even exerted beneficial effects, which are opposite to major findings that alcohol consumption protects from myocardial infarction revealed in the INTERHEART China study. Given the unique geographical (Wu dialect area) and yellow rice wine (homemade wine) cultural features of Suzhou City with a permanent population of around 6.5 million and the largest tertiary medical center where our study has been conducted, our findings have likewise pointed to the possible presence of Suzhou Paradox although we have to admit the small event number in terms of alcohol abstainers in both case and control groups. An independent study is warranted for further verifying our findings.

A mounting body of evidence has established that LSA^12^,^13^,^14^ and high Lp(a) concentrations^10^,^40^,^41^,^42^,^43^ were 2 important risk factors for CADs both in Chinese and in Caucasians. Atherosclerosis has been viewed as an inflammatory disease. Albumin has been described as an important extracellular antioxidant for binding metals and scavenging peroxyl radicals^44^. Also, serum albumin has been postulated to inhibit endothelia apoptosis^45^ and stabilize endothelium-derived relaxing factor based on *in vitro* and animal experiments^46^. Additionally, serum albumin acts as a shuttle to enhance cholesterol efflux from cells, perhaps, mitigating the process of atherosclerosis^47^. In sum, many putative mechanisms have been identified by which LSA concentration may, separately, or in combination, play a role in the initiation of atherosclerosis, ultimately leading to the onset of AMI.

However, little has been done to explore the risk burdens conferred by these 2 risk factors in patients with CAD. In the current study, Lp(a) was significantly associated with first incident AMI, accounting for on average 19% of PAR, in both sexes in oldest 2 age categories; LSA concentrations were significantly associated with first incident AMI, accounting for on average 41% of PAR, in both sexes across all age categories. Greater risk burdens were seen with both Lp (a) and LSA concentrations in women than in men. The risk burdens of Lp (a) and LSA were similar (~19% and ~39%, respectively) across all age categories in men. By contrast, the risk burdens of Lp (a) and LSA were greater in oldest 2 age categories than in youngest age category in women. Any PAR calculation in combination with LSA, Lp (a) or both, will increase substantially. LSA and Lp (a) together accounted for more than 50% of PAR of first incident AMI in both sexes in most age categories. Lp (a) has been proposed a marker for residual cardiovascular risk^15^. LSA, reflecting a general condition of a patient rather than a specific indicator (e.g. LDL-C in abnormal LDL-C metabolism, troponin I in myocardial injuries), may be a better target for global CAD risk intervention so as to further reduce the incidence of AMI^48^.

### Strengths and limitations

The strengths and limitations merit careful considerations: this is the largest cross-sectional study to date reporting the risk burdens of individual risk factors incorporating LSA and Lp(a) for first incident AMI in Chinese Han ethnic hospital-based population. We have used LDL-C levels as a marker of dyslipidemia, a primary therapy target recommended by Guidelines at home and abroad, facilitating comparison of our findings with other countries. We have examined modifiable risk factors incorporating LSA and Lp (a) simultaneously, which made us for the first time know the risk burdens of LSA and Lp (a) in the context of conventional modifiable risk factors. As high as 28% of non-CAD controls underwent coronary angiography, a gold standard for diagnosing CAD, enabling us to group individuals more accurately as compared with population-based studies, where apparently “healthy” individuals may be actually with CAD. The current study has considered possible presence of multicollinearity among covariates in model fitting. Thus, the ratio of apo A/apo B, apo B, apo A, and TC were removed from the model fitting. Confounding factors inherent in cross-sectional design are unavoidable. A total of 1334 patients were excluded from the current study due to lack of Lp (a) exams, which may bias our results. However, prescription of Lp (a) exams was at discretion of physicians, not focusing on a specific population. Therefore, our findings are impossible to be changed materially even after inclusion of these individuals for final analysis. Exclusion of prior CAD or ischemic heart disease in the current study minimizes the effects of drug administration or misclassification on the association of risk factor exposure with first incident AMI.

The representation of this sample has remained a concern as the participants in the current study were hospital-based. We consecutively included all the participants for potential analysis admitted between Jan 1, 2010 and Dec 31, 2013, and obtained studied participants for final analysis by excluding the ineligible according to uniform exclusion criteria. By doing so, we maximized the representation of the sample used in the current study. Interestingly, our hospital-based study reported a similar PAR for smoking (36% vs 31%) to that reported in Chinese/HK population in the INTERHEART study^8^. Therefore, the representation of the current study may be reasonable although we have to admit that calculation risk burdens using a cohort study will be better. Our findings revealed in the current study should be cautiously applied to the general population.

## Conclusions

Modifiable risk factors incorporating LSA and Lp (a) have accounted for an overwhelmingly large proportion of the risk of first incident AMI, indicating that most AMI is preventable. The effects of these risk factors are different in women and men, across age categories, entailing tailored prevention strategies for different populations. After multivariable adjustment, Lp(a) and LSA have accounted for 19% and 41%, respectively, and together for more than a half (54%) of PAR for first incident AMI. Risk factor intervention strategies incorporating Lp(a) and LSA may be beneficial for reduction of incidence and mortality of CVDs from a new angle.

## Additional Information

**How to cite this article**: Yang, Q. *et al*. Risk burdens of modifiable risk factors incorporating lipoprotein (a) and low serum albumin concentrations for first incident acute myocardial infarction. *Sci. Rep.* **6**, 35463; doi: 10.1038/srep35463 (2016).

## Figures and Tables

**Table 1:** Demographic data of first incident AMI and non-CAD controls.

Characteristics	Missing	Non-CAD	First AMI	P values
N	n (%)	6125	1522	
**Demographic data**				
Male n (%)		3165(51.67)	1229(80.75)	<0.0001
Age(IQR), yr		61(21.00)	64(18.00)	<0.0001
Height(IQR), cm	447(5.85)	163(12.00)	166(10.00)	<0.0001
Body weight(IQR), kg	812(10.62)	64(16.00)	66(13.00)	<0.0001
BMI(IQR), kg/m2		23.88(4.12)	24.09(1.36)	0.0020
Marriage n (%)	14(0.77)			<0.0001
Divorced		1(0.02)	3(0.20)	
Married		5932(96.85)	1492(98.03)	
Unmarried		144(2.35)	15(0.99)	
Widowed		48(0.78)	12(0.79)	
**Vital signs**				
Sbp(IQR), mmHg	42(0.55)	130(22.00)	123(29.00)	<0.0001
Dbp(IQR), mmHg	47(0.61)	80(15.00)	75(17.00)	
Heart rate(IQR), bpm	54(0.71)	72(19.00)	75(22.00)	<0.0001
**Life styles**				
Smoking n (%)	276(3.61)			<0.0001
Never		4403(71.89)	578(37.98)	
Past		430(7.02)	123(8.08)	
Current		1292(21.09)	821(53.94)	
Drinking n (%)	278(3.64)			<0.0001
Never		5178(84.54)	1108(72.80)	
Past		122(1.99)	35(2.30)	
Current		825(13.47)	379(24.90)	
**Past history**				
HT n (%)	0(0)	3392(55.38)	926(60.84)	<0.0001
DM n (%)	0(0)	753(12.29)	346(22.73)	<0.0001
Is-stroke n (%)	0(0)	362(5.91)	80(5.26)	0.3220
He-stroke n (%)	0(0)	17(0.28)	6(0.39)	0.4720
**Blood analysis**				
Total protein (IQR), g/L	49(0.64)	67.80 (8.50)	64.80 (7.70)	<0.0001
Alb (IQR), g/L	49(0.64)	41.90 (6.00)	39.50 (5.80)	<0.0001
Creatinin (IQR), μmol/L	50(0.65)	73.00 (25.00)	78.00 (24.10)	<0.0001
ALT (IQR), U/L	49(0.64)	19.00 (15.00)	40.20 (39.80)	<0.0001
AST (IQR), U/L	49(0.64)	22.40 (9.00)	123.50 (245.00)	<0.0001
Hemoglobin (IQR), g/L	341(4.46)	134.00 (21.00)	135.00 (20.00)	0.4794
**Lipid profiles**				
TC (IQR), mmol/L	0(0)	4.12 (1.28)	4.14 (1.26)	0.0076
TG (IQR), mmol/L)	0(0)	1.17 (0.92)	1.23 (0.97)	0.0037
Lp(a) (IQR), mg/dL	0(0)	7.40 (13.10)	11.10 (19.20)	<0.0001
Apo A (IQR), g/L	0(0)	1.31 (0.26)	1.21 (0.22)	<0.0001
Apo B (IQR), g/L	0(0)	0.89 (0.28)	0.93 (0.29)	<0.0001
Apo A1/B (IQR)	0(0)	1.40 (0.50)	1.20 (0.40)	<0.0001
HDL-C (IQR), mmol/L	0(0)	1.12 (0.36)	1.01 (0.28)	<0.0001
LDL-C (IQR), mmol/L)	0(0)	2.42 (0.94)	2.59 (1.07)	<0.0001
**Medications n (%)**				
Aspirin	0(0)	1941 (31.69)	1368 (89.88)	<0.0001
Clopidogrel	0(0)	227 (3.71)	1364 (89.62)	<0.0001
ACEI or ARB	0(0)	2567 (41.91)	1384 (90.93)	<0.0001
Beta blockers	0(0)	2296 (37.49)	1182 (77.66)	<0.0001
Calcium channel blockers	0(0)	1630 (26.61)	102 (6.70)	<0.0001
Statins	0(0)	1458 (23.80)	1458 (92.31)	<0.0001
Nitrates	0(0)	454 (7.41)	407 (26.74)	<0.0001
**Imaging**	0(0)			
CAG (%)		1691(27.61)	1385(91.00)	<0.0001

BMI indicated body mass index; sbp, systolic blood pressure; dbp, diastolic blood pressure; bpm, beat per minute; HT, primary hypertension; DM, diabetes mellitus; is-stroke, ischemic stroke; he-stroke, hemorrhagic stroke; alb, albumin; ALT, alanine transferase; AST, aspartic tranferase; TC, total cholesterol; TG, triglycerides; Lp(a), lipoprotein (a); apo A, apolipoprotein A1; apo B, apolipoprotien B; HDL-C, high density lipoprotein cholesterol; LDL-C, low density lipoprotein cholesterol; ACEI, angiotensin converting enzyme inhibitor; ARB, angiotension-II receptor blocker; CAG, coronary angiography.

**Table 2:** Risk of first incident AMI associated with risk factors in the overall population.

Risk factors	Exposed cases n (%)	Exposed controls n (%)	cOR(95%CI)	cPAR(95%CI)	¶OR(95%CI)	¶PAR(95%CI)	§OR(95%CI)	§PAR(95%CI)
**Sex (Male%)**	1229(80.75)	3165(51.67)	3.92(3.42–4.50)	0.55(0.50–0.59)				
**Age, y, subtotal**	1141(74.97)	4045(66.04)		0.22(0.16–0.28)				
Middle/bottom	539(35.41)	2040(33.31)	1.44(1.25–1.67)	0.09(0.06–0.13)				
Top/bottom	602(39.55)	2005(32.73)	1.64(1.42–1.89)	0.13(0.09–0.17)				
**Is-stroke (%)**	80(5.26)	362(5.91)	0.88(0.69–1.13)	−0.01(−0.02–0.01)	0.71(0.55–0.92)	−0.02(−0.03–0.00)	0.57(0.43–0.75)	−0.02(−0.03–0.01)
HT (%)	926(60.84)	3392(55.38)	1.25(1.12–1.40)	0.10(0.05–0.15)	1.10(0.97–1.24)	0.04(−0.01–0.10)	1.05(0.92–1.21)	0.02(−0.03–0.07)
DM (%)	346(22.73)	753(12.29)	2.10(1.82–2.42)	0.10(0.08–0.12)	2.04(1.76–2.37)	0.09(0.07–0.11)	2.10(1.77–2.48)	0.08(0.06–0.10)
**Drinking (%), subtotal**	414(27.20)	947(15.46)		0.11(0.09–0.14)		0.05(0.03–0.08)		−0.02(−0.04–0.01)
Past	35(2.30)	122(1.99)	1.34(0.92–1.96)	0.00(0.00–0.01)	0.74(0.50–1.09)	−0.01(−0.01–0.00)	0.64(0.41–0.99)	−0.01(−0.01–0.00)
Current	379(24.90)	825(13.47)	2.15(1.87–2.46)	0.11(0.09–0.13)	1.44(1.24–1.67)	0.06(0.03–0.08)	0.94(0.79–1.12)	−0.01(−0.03–0.02)
**Smoking (%), subtotal**	944(62.02)	1722(28.11)		0.42(0.38–0.45)		0.34(0.30–0.38)		0.31(0.26–0.35)
Past	123(8.08)	430(7.02)	2.18(1.75–2.71)	0.04(0.03–0.05)	1.29(1.02–1.63)	0.01(0.00–0.03)	1.30(1.00–1.68)	0.01(0.00–0.03)
Current	821(53.94)	1292(21.09)	4.84(4.28–5.48)	0.38(0.35–0.41)	3.65(3.14–4.25)	0.33(0.29–0.36)	3.57(3.01–4.24)	0.29(0.26–0.33)
**BMI(kg/m**^**2**^**), subtotal**	1196(78.58)	4077(66.56)		0.31(0.25–0.37)		0.27(0.21–0.33)		0.21(0.14–0.27)
Middle/bottom	859(56.44)	2038(33.27)	2.65(2.30–3.05)	0.30(0.26–0.34)	2.52(2.18–2.92)	0.28(0.24–0.32)	2.38(2.03–2.80)	0.23(0.19–0.27)
Top/bottom	337(22.14)	2039(33.29)	1.04(0.88–1.22)	0.01(−0.02–0.04)	0.96(0.81–1.13)	−0.01(−0.04–0.02)	0.86(0.71–1.04)	−0.03(−0.06–0.01)
**Alb, g/L, subtotal**	1300(85.41)	4108(67.07)		0.50(0.44–0.56)		0.47(0.41–0.53)		0.41(0.34–0.47)
Middle/top	446(29.30)	2052(33.50)	1.97(1.66–2.35)	0.13(0.10–0.16)	1.93(1.62–2.31)	0.12(0.09–0.15)	1.76(1.45–2.13)	0.10(0.06–0.13)
Bottom/top	854(56.11)	2056(33.57)	3.77(3.22–4.43)	0.37(0.33–0.41)	3.51(2.96–4.17)	0.35(0.31–0.39)	3.47(2.86–4.20)	0.31(0.27–0.36)
**HDL-C, mmol/L, subtotal**	1238(81.34)	4094(66.84)		0.38(0.32–0.44)		0.27(0.20–0.34)		0.16(0.09–0.23)
Middle/top	465(30.55)	1988(32.46)	1.67(1.43–1.96)	0.11(0.07–0.14)	1.38(1.17–1.63)	0.07(0.03–0.10)	1.23(1.02–1.47)	0.04(0.00–0.07)
Bottom/top	773(50.79)	2106(34.38)	2.62(2.26–3.05)	0.28(0.24–0.32)	1.96(1.68–2.29)	0.20(0.16–0.25)	1.58(1.32–1.89)	0.12(0.08–0.17)
**Lp(a), mg/L, subtotal**	1185(77.86)	4042(65.99)		0.30(0.24–0.36)		0.28(0.22–0.34)		0.19(0.13–0.26)
Middle/bottom	493(32.39)	2006(32.75)	1.52(1.31–1.77)	0.10(0.06–0.13)	1.48(1.27–1.73)	0.09(0.05–0.12)	1.34(1.13–1.58)	0.06(0.02–0.09)
Top/bottom	692(45.47)	2036(33.24)	2.10(1.82–2.43)	0.21(0.17–0.24)	2.13(1.83–2.47)	0.20(0.16–0.23)	1.80(1.53–2.13)	0.14(0.10–0.18)
**LDL-C, mmol/L, subtotal**	1139(74.84)	4067(66.40)		0.21(0.15–0.27)		0.25(0.19–0.31)		0.25(0.19–0.30)
Middle/bottom	483(31.73)	2046(33.40)	1.27(1.09–1.47)	0.06(0.02–0.09)	1.37(1.18–1.60)	0.07(0.04–0.10)	1.47(1.24–1.74)	0.07(0.04–0.10)
Top/bottom	656(43.10)	2021(33.00)	1.74(1.52–2.01)	0.16(0.12–0.19)	2.05(1.77–2.37)	0.18(0.14–0.22)	2.27(1.91–2.69)	0.17(0.14–0.21)
**TG, mmol/L, subtotal**	1077(70.76)	4044(66.02)		0.11(0.05–0.18)		0.15(0.09–0.21)		0.05(−0.01–0.12)
Middle/bottom	538(35.35)	2022(33.01)	1.24(1.08–1.43)	0.06(0.02–0.09)	1.32(1.14–1.53)	0.07(0.03–0.10)	1.12(0.95–1.32)	0.02(−0.01–0.06)
Top/bottom	539(35.41)	2022(33.01)	1.25(1.08–1.43)	0.06(0.02–0.09)	1.41(1.21–1.63)	0.08(0.05–0.12)	1.16(0.97–1.39)	0.03(−0.01–0.07)

All continuous variables presented here were divided into thirds. Middle indicated the middle third in the measurement of the continuous variable; top, top third; and bottom, bottom third. A cOR indicated crude OR without any adjustment for risk factors; ¶OR, partially adjusted OR after adjustment for age and sex only; §OR, fully adjusted OR after adjustment for Lp(a), drinking, smoking, HT, DM, BMI, LDL-C, HDL-C, TG, ALB, ischemic stroke, exam year, creatinine, and hemorrhagic stroke plus age and sex. OR indicated odds ratio; PAR, population attributable risk. Other abbreviations as presented in Table 1.

**Table 3:** PARs combination calculation for risk factors significantly associated with first incident AMI in overall population and stratified by sex and age.

Populations	PARs Combination for risk factors	PAR (95% CI)
Overall	Smoking, LDL-C, BMI, HDL-C & DM	0.76(0.71–0.81)
	LSA addition	0.89(0.86–0.92)
	Lp(a) addition	0.83(0.78–0.87)
	LSA & Lp(a) addition	0.92(0.90–0.94)
	LSA & Lp(a)	0.54(0.48–0.61)
Women	LDL-C,DM,BMI,TG & HT	0.72(0.61–0.83)
	LSA addition	0.87(0.80–0.94)
	Lp(a) addition	0.81(0.72–0.90)
	LSA & Lp(a) addition	0.91(0.86–0.96)
	LSA & Lp(a)	0.65(0.52–0.78)
Men	Smoking,BMI,LDL-C,DM & HDL-C	0.80(0.75–0.85)
	LSA addition	0.91(0.88–0.94)
	Lp(a) addition	0.85(0.81–0.90)
	LSA & Lp(a) addition	0.93(0.91–0.96)
	LSA & Lp(a)	0.52(0.45–0.59)
Women_age1	TG & BMI	0.85(0.66–1.03)
	LSA addition	0.90(0.77–1.02)
Women_age2	DM, TG & LDL-C	0.70(0.49–0.91)
	LSA addition	0.85(0.72–0.99)
	Lp(a) addition	0.84(0.71–0.98)
	LSA & Lp(a) addition	0.92(0.84–1.01)
	LSA & Lp(a)	0.72(0.55–0.89)
Women_age3	BMI, LDL-C & DM	0.55(0.37–0.73)
	LSA addition	0.80(0.66–0.94)
	Lp(a) addition	0.67(0.50–0.84)
	LSA & Lp(a) addition	0.86(0.75–0.97)
	LSA & Lp(a)	0.63(0.40–0.87)
Men_age1	Smoking, BMI, LDL-C, DM & HDL-C	0.83(0.73–0.92)
	LSA addition	0.91(0.85–0.96)
	Lp(a) addition	0.87(0.80–0.95)
	LSA & Lp(a) addition	0.93(0.89–0.98)
	LSA & Lp(a)	0.48(0.37–0.59)
Men_age2	Smoking, DM, BMI, LDL-C & HDL-C	0.83(0.74–0.91)
	LSA addition	0.92(0.88–0.97)
	Lp(a) addition	0.87(0.80–0.94)
	LSA & Lp(a) addition	0.94(0.90–0.98)
	LSA & Lp(a)	0.48(0.35–0.62)
Men_age3	Smoking, BMI, LDL-C, DM & HDL-C	0.76(0.67–0.85)
	LSA addition	0.87(0.80–0.94)
	Lp(a) addition	0.83(0.75–0.91)
	LSA & Lp(a) addition	0.91(0.86–0.96)
	LSA & Lp(a)	0.49(0.31–0.67)

PAR combination calculations were conducted only for those risk factors significantly associated with first incident AMI in corresponding population or subgroups. The overall population taken as an example, Smoking, LDL-C, BMI, HDL-C & DM indicated the combination calculation of the PAR for these 5 risk factors; LSA or Lp(a) addition indicated combination calculation of the PAR for the 5 risk factors as well as LSA or Lp(a) separately; LSA & Lp(a) addition indicated calculation of the PAR for the 5 risk factors as well as LSA and Lp(a) together. LSA & Lp(a) indicated combination calculation of the PAR for these 2 risk factors. The rest populations or stratifications could be done in the same way. Age1–3 indicated tirtiles of the continuous age: ≤55 y, 55~67 y and >67 y. Abbreviations presented as in Table 1.

**Table 4:** Risk of first incident AMI associated with risk factors grouped by sex.

Risk factors	Women				Men			
	Exposed cases n (%)	Exposed controls n (%)	§OR(95%CI)	§PAR(95%CI)	Exposed cases n (%)	Exposed controls n (%)	§OR(95%CI)	§PAR(95%CI)
**Is-stroke (%)**	18(6.14)	159(5.37)	0.62(0.36–1.06)	−0.03(−0.05–0.00)	62(5.04)	203(6.41)	0.56(0.40–0.78)	−0.02(−0.03–0.01)
HT (%)	212(72.35)	1620(54.73)	1.38(1.02–1.87)	0.17(0.01–0.32)	714(58.10)	1772(55.99)	0.98(0.83–1.15)	−0.01(−0.06–0.05)
DM (%)	82(27.99)	372(12.57)	2.13(1.56–2.90)	0.12(0.07–0.18)	264(21.48)	381(12.04)	2.05(1.68–2.51)	0.07(0.05–0.09)
**Drinking (%), subtotal**	9(3.07)	47(1.59)		0.01(0.00–0.03)	405(32.95)	900(28.44)		−0.02(−0.06–0.01)
Past	1(0.34)	3(0.10)	2.99(0.18–49.66)		34(2.77)	119(3.76)	0.63(0.4–0.98)	−0.01(−0.02–0.00
Current	8(2.73)	44(1.49)	2.11(0.85–5.20)	0.01(0.00–0.03)	371(30.19)	781(24.68)	0.91(0.76–1.09)	−0.02(−0.05–0.02)
**Smoking (%), subtotal**	11(3.75)	77(2.60)		0.00(−0.02–0.02)	933(75.92)	1645(51.97)		0.39(0.33–0.44)
Past	2(0.68)	17(0.57)	0.87(0.19–4.04)	0.00(−0.01–0.01)	121(9.85)	413(13.05)	1.39(1.07–1.81)	0.02(0.00–0.04)
Current	9(3.07)	60(2.03)	1.07(0.46–2.44)	0.00(−0.02–0.02)	812(66.07)	1232(38.93)	3.72(3.11–4.45)	0.37(0.32–0.41)
**BMI(kg/m**^**2**^**), subtotal**	218(74.40)	1841(62.20)		0.20(0.06–0.35)	978(79.58)	2236(70.65)		0.21(0.13–0.28)
Middle/bottom	168(57.34)	982(33.18)	2.08(1.53–2.82)	0.26(0.15–0.36)	691(56.22)	1056(33.36)	2.47(2.04–2.99)	0.23(0.18–0.27)
Top/bottom	50(17.06)	859(29.02)	0.74(0.50–1.10)	−0.05(−0.12–0.02)	287(23.35)	1180(37.28)	0.90(0.72–1.12)	−0.02(−0.05–0.02)
**Alb, g/L, subtotal**	252(86.01)	1992(67.30)		0.48(0.34–0.63)	1048(85.27)	2116(66.86)		0.39(0.32–0.46)
Middle/top	64(21.84)	1043(35.24)	1.34(0.88–2.04)	0.05(−0.02–0.12)	382(31.08)	1009(31.88)	1.89(1.52–2.35)	0.11(0.07–0.14)
Bottom/top	188(64.16)	949(32.06)	3.80(2.57–5.63)	0.43(0.33–0.53)	666(54.19)	1107(34.98)	3.37(2.70–4.22)	0.29(0.24–0.33)
**HDL-C, mmol/L, subtotal**	197(67.24)	1709(57.74)		0.05(−0.09–0.20)	1041(84.70)	2385(75.36)		0.20(0.12–0.29)
Middle/top	95(32.42)	939(31.72)	1.05(0.76–1.46)	0.01(−0.07–0.10)	370(30.11)	1049(33.14)	1.33(1.07–1.66)	0.05(0.01–0.09)
Bottom/top	102(34.81)	770(26.01)	1.17(0.83–1.64)	0.04(−0.05–0.13)	671(54.60)	1336(42.21)	1.78(1.43–2.21)	0.15(0.10–0.21)
**Lp(a), mg/L, subtotal**	236(80.55)	1990(67.23)		0.30(0.14–0.45)	949(77.22)	2052(64.83)		0.17(0.11–0.24)
Middle/bottom	96(32.76)	949(32.06)	1.56(1.09–2.24)	0.10(0.02–0.18)	397(32.30)	1057(33.40)	1.28(1.06–1.56)	0.05(0.01–0.08)
Top/bottom	140(47.78)	1041(35.17)	1.89(1.33–2.67)	0.19(0.10–0.29)	552(44.91)	995(31.44)	1.80(1.49–2.17)	0.13(0.09–0.17)
**LDL-C, mmol/L, subtotal**	236(80.55)	2029(68.55)		0.35(0.20–0.49)	903(73.47)	2038(64.39)		0.21(0.15–0.28)
Middle/bottom	99(33.79)	971(32.80)	1.78(1.23–2.56)	0.13(0.05–0.21)	384(31.24)	1075(33.97)	1.37(1.13–1.66)	0.06(0.02–0.09)
Top/bottom	137(46.76)	1058(35.74)	2.15(1.50–3.09)	0.22(0.13–0.31)	519(42.23)	963(30.43)	2.26(1.85–2.75)	0.16(0.12–0.20)
**TG, mmol/L, subtotal**	218(74.40)	1932(65.27)		0.18(0.03–0.33)	859(69.89)	2112(66.73)		0.01(−0.06–0.08)
Middle/bottom	117(39.93)	973(32.87)	1.50(1.08–2.10)	0.11(0.02–0.20)	421(34.26)	1049(33.14)	0.99(0.81–1.20)	0.00(−0.04–0.04)
Top/bottom	101(34.47)	959(32.40)	1.30(0.90–1.88)	0.07(−0.02–0.16)	438(35.64)	1063(33.59)	1.08(0.87–1.33)	0.01(−0.03–0.06)

Abbreviations presented as in Table 1. §OR indicated fully adjusted OR after adjustment for risk factors mentioned in this table.

**Table 5:** Risk of first incident AMI associated with risk factors stratified by sex and age.

Risk factors for women	≤55 y		55 ~ 67 y		>67 y	
	§OR(95%CI)	§PAR(95%CI)	§OR(95%CI)	§PAR(95%CI)	§OR(95%CI)	§PAR(95%CI)
**Is-stroke**	**NA**	NA	0.89(0.32–2.48)	−0.01(−0.06–0.04)	0.51(0.26–1.02)	−0.04(−0.08–0.01)
HT	1.78(0.79–4.01)	0.21(−0.07–0.49)	1.50(0.89–2.52)	0.19(−0.05–0.43)	1.11(0.73–1.71)	0.06(−0.18–0.30)
DM	2.41(0.73–7.96)	0.08(−0.06–0.23)	3.18(1.88–5.36)	0.21(0.10–0.32)	1.83(1.20–2.81)	0.10(0.02–0.17)
**Drinking, subtotal**		0.02(−0.03–0.06)		0.02(−0.02–0.05)		0.01(−0.01–0.03)
Past	NA	NA	NA	NA	5.03(0.15–166.76)	0.00(0.00–0.01)
Current	2.49(0.24–25.71)	0.02(−0.03–0.06)	2.18(0.49–9.83)	0.02(−0.02–0.05)	1.85(0.48–7.12)	0.01(−0.01–0.03)
**Smoking, subtotal**		0.01(−0.03–0.05)		0.01(−0.02–0.04)		0.00(−0.03–0.04)
Past	NA	NA	NA	NA	1.33(0.27–6.67)	0.00(−0.01–0.02)
Current	1.98(0.19–20.43)	0.01(−0.03–0.05)	1.49(0.34–6.56)	0.01(−0.02–0.04)	1.02(0.32–3.21)	0.00(−0.03–0.03)
**BMI(kg/m**^**2**^**), subtotal**		0.44(−0.02–0.89)		−0.04(−0.37–0.28)		0.25(0.08–0.43)
Middle/bottom	3.20(1.1–9.33)	0.39(0.11–0.68)	1.19(0.68–2.10)	0.06(−0.13–0.24)	2.47(1.65–3.68)	0.32(0.19–0.45)
Top/bottom	1.25(0.36–4.38)	0.05(−0.21–0.30)	0.69(0.37–1.29)	−0.10(−0.28–0.08)	0.57(0.31–1.04)	−0.07(−0.13–0.00)
**Alb, g/L, subtotal**		0.30(−0.07–0.66)		0.47(0.25–0.69)		0.49(0.23–0.76)
Middle/top	1.11(0.41–3.01)	0.02(−0.21–0.25)	1.38(0.71–2.70)	0.07(−0.07–0.20)	1.34(0.67–2.70)	0.04(−0.06–0.14)
Bottom/top	3.29(1.26–8.62)	0.27(0.05–0.49)	4.87(2.57–9.24)	0.40(0.26–0.54)	3.2(1.69–6.09)	0.45(0.26–0.64)
**HDL-C, mmol/L, subtotal**		−0.10(−0.68–0.48)		−0.03(−0.29–0.24)		0.09(−0.09–0.28)
Middle/top	0.54(0.19–1.51)	−0.18(−0.51–0.16)	0.89(0.50–1.58)	−0.03(−0.2–0.13)	1.26(0.81–1.96)	0.05(−0.05–0.16)
Bottom/top	1.27(0.46–3.5)	0.08(−0.24–0.39)	1.02(0.55–1.91)	0.01(−0.14–0.16)	1.16(0.73–1.84)	0.04(−0.08–0.15)
**Lp(a), mg/L, subtotal**		0.15(−0.38–0.69)		0.42(0.18–0.67)		0.25(0.03–0.46)
Middle/bottom	2.17(0.84–5.64)	0.24(−0.05–0.52)	1.50(0.75–2.99)	0.08(−0.05–0.20)	1.46(0.90–2.39)	0.09(−0.02–0.20)
Top/bottom	0.75(0.26–2.16)	−0.08(−0.41–0.25)	3.20(1.68–6.09)	0.35(0.19–0.51)	1.70(1.06–2.73)	0.16(0.02–0.30)
**LDL-C, mmol/L, subtotal**		0.57(0.19–0.95)		0.39(0.11–0.67)		0.30(0.11–0.48)
Middle/bottom	2.95(0.89–9.77)	0.27(0.02–0.52)	2.04(1.02–4.06)	0.16(0.01–0.30)	1.59(0.98–2.59)	0.10(0.00–0.20)
Top/bottom	3.30(0.96–11.32)	0.30(0.06–0.53)	2.18(1.11–4.30)	0.23(0.05–0.41)	2.04(1.27–3.28)	0.20(0.07–0.32)
**TG, mmol/L, subtotal**		0.69(0.35–1.03)		0.33(0.03–0.64)		0.05(−0.15–0.24)
Middle/bottom	4.14(1.11–15.51)	0.31(0.07–0.54)	2.19(1.12–4.26)	0.21(0.04–0.38)	1.12(0.73–1.73)	0.03(−0.09–0.15)
Top/bottom	5.27(1.33–20.93)	0.38(0.15–0.61)	1.57(0.77–3.21)	0.12(−0.06–0.31)	1.08(0.66–1.76)	0.02(−0.09–0.12)
**Risk factors for men**						
**Is-stroke**	0.35(0.09–1.27)	−0.01(−0.01–0.00)	0.65(0.35–1.19)	−0.01(−0.03–0.00)	0.53(0.34–0.80)	−0.04(−0.07-−0.02)
HT	1.25(0.94–1.67)	0.06(−0.02–0.13)	1.02(0.78–1.34)	0.01(−0.08–0.09)	0.73(0.55–0.96)	−0.13(−0.25-−0.01)
DM	2.00(1.33–2.99)	0.06(0.02–0.09)	2.39(1.70–3.35)	0.08(0.05–0.11)	1.87(1.35–2.59)	0.07(0.03–0.10)
**Drinking, subtotal**		0.01(−0.07–0.08)		−0.09(−0.15-−0.03)		0.01(−0.03–0.05)
Past	0.64(0.2–2.04)	0.00(−0.01–0.01)	0.32(0.13–0.77)	−0.02(−0.03–0.00)	0.90(0.49–1.65)	0.00(−0.02–0.01)
Current	1.05(0.78–1.42)	0.01(−0.06–0.08)	0.67(0.50–0.89)	−0.07(−0.13-−0.02)	1.12(0.79–1.59)	0.01(−0.03–0.05)
**Smoking, subtotal**		0.47(0.36–0.58)		0.41(0.32–0.51)		0.28(0.20–0.36)
Past	2.97(1.51–5.83)	0.03(0.01–0.05)	0.99(0.61–1.61)	0.00(−0.03–0.03)	1.34(0.92–1.94)	0.03(−0.01–0.07)
Current	3.57(2.53–5.05)	0.44(0.33–0.55)	3.99(2.93–5.45)	0.41(0.33–0.49)	3.53(2.61–4.76)	0.25(0.19–0.31)
**BMI(kg/m**^**2**^**), subtotal**		0.27(0.09–0.45)		0.19(0.06–0.31)		0.19(0.09–0.29)
Middle/bottom	2.98(1.98–4.48)	0.27(0.18–0.37)	2.32(1.66–3.25)	0.2(0.12–0.27)	2.41(1.80–3.22)	0.22(0.15–0.29)
Top/bottom	0.99(0.63–1.53)	0.00(−0.10–0.10)	0.95(0.65–1.37)	−0.01(−0.08–0.06)	0.73(0.50–1.08)	−0.03(−0.08–0.01)
**Alb, g/L, subtotal**		0.34(0.24–0.44)		0.38(0.25–0.50)		0.34(0.14–0.53)
Middle/top	2.21(1.6–3.05)	0.16(0.10–0.23)	1.70(1.16–2.48)	0.10(0.03–0.16)	1.45(0.88–2.41)	0.04(−0.01–0.10)
Bottom/top	3.58(2.46–5.21)	0.17(0.12–0.23)	3.62(2.48–5.30)	0.28(0.21–0.35)	2.37(1.48–3.79)	0.30(0.15–0.44)
**HDL-C, mmol/L, subtotal**		0.11(−0.06–0.28)		0.24(0.09–0.38)		0.24(0.10–0.37)
Middle/top	0.93(0.62–1.41)	−0.01(−0.08–0.06)	1.57(1.07–2.30)	0.08(0.01–0.14)	1.50(1.04–2.17)	0.07(0.01–0.13)
Bottom/top	1.50(1.02–2.21)	0.12(0.01–0.24)	1.90(1.30–2.77)	0.16(0.07–0.25)	1.95(1.35–2.82)	0.17(0.08–0.26)
**Lp(a), mg/L, subtotal**		0.18(0.07–0.29)		0.14(0.03–0.24)		0.19(0.07–0.32)
Middle/bottom	1.29(0.91–1.82)	0.04(−0.02–0.11)	1.11(0.80–1.54)	0.02(−0.04–0.08)	1.42(1.01–2.02)	0.07(0.00–0.14)
Top/bottom	1.9(1.36–2.67)	0.13(0.06–0.20)	1.76(1.28–2.42)	0.12(0.05–0.18)	1.76(1.25–2.48)	0.12(0.05–0.20)
**LDL-C, mmol/L, subtotal**		0.25(0.10–0.39)		0.21(0.11–0.31)		0.19(0.10–0.28)
Middle/bottom	1.41(0.96–2.08)	0.06(−0.01–0.13)	1.38(1.00–1.91)	0.06(0.00–0.12)	1.38(1.01–1.88)	0.06(0.00–0.11)
Top/bottom	2.16(1.48–3.16)	0.19(0.10–0.28)	2.29(1.63–3.21)	0.15(0.09–0.21)	2.38(1.71–3.33)	0.14(0.08–0.19)
**TG, mmol/L, subtotal**		0.06(−0.13–0.24)		0.05(−0.06–0.17)		−0.03(−0.12–0.06)
Middle/bottom	0.98(0.64–1.49)	0.00(−0.08–0.07)	1.16(0.83–1.61)	0.03(−0.04–0.09)	0.90(0.67–1.22)	−0.02(−0.08–0.04)
Top/bottom	1.24(0.82–1.87)	0.06(−0.06–0.18)	1.13(0.80–1.61)	0.02(−0.04–0.09)	0.91(0.62–1.32)	−0.01(−0.05–0.03)

Age was divided into thirds with the upper limit being inclusive. Abbreviations as in table. §OR indicated fully adjusted OR after adjustment for risk factors mentioned in table. NA indicated “not available” because of low number of study subjects exposed to the risk factor.
